# Primary care management of hidradenitis suppurativa: a cross-sectional survey of UK GPs

**DOI:** 10.3399/BJGPO.2021.0051

**Published:** 2021-09-29

**Authors:** Fiona Collier, Rachel Howes, Jeremy Rodrigues, Kim S Thomas, Paul Leighton, John R Ingram

**Affiliations:** 1 Portfolio GP and GP with Special Interest in Dermatology, NHS Forth Valley, Larbert, UK; 2 Department of Plastic Surgery, Salisbury Hospital, Salisbury NHS Foundation Trust, Salisbury, UK; 3 Department of Plastic Surgery, Stoke Mandeville Hospital, Buckinghamshire Healthcare NHS Trust, Amersham, UK; 4 Nuffield Department of Orthopaedics, Rheumatology and Musculoskeletal Sciences, University of Oxford, Oxford, UK; 5 Centre of Evidence Based Dermatology, School of Medicine, University of Nottingham, Nottingham, UK; 6 Department of Dermatology & Academic Wound Healing, Division of Infection and Immunity, Cardiff University, Cardiff, UK

**Keywords:** hidradenitis suppurativa, primary health care, dermatitis

## Abstract

**Background:**

Hidradenitis suppurativa (HS) is a chronic inflammatory skin disease that causes painful discharging nodules and skin tunnels. HS has associations with several systemic diseases, including cardiovascular disease (CVD), anxiety, and depression. High levels of chronic morbidity suggest an important role for primary care. However, little evidence exists regarding current management of HS and its comorbidities in UK general practice.

**Aim:**

To describe current practice among UK GPs in treating and referring people with HS.

**Design & setting:**

A web-based survey was circulated to UK Primary Care Dermatology Society (PCDS) members and GPs in Forth Valley, Scotland.

**Method:**

Survey responses were analysed with descriptive statistics.

**Results:**

A total of 134 UK GPs completed the survey. Seventy per cent (*n* = 94) saw at least one patient with HS in the previous month. Ninety-four per cent (*n* = 125/133) reported confidence in diagnosis, and 89% (*n* = 120/134) in initial treatment of HS. Most GPs initiated topical treatments and extended courses of oral antibiotic for HS, and many gave advice on adverse lifestyle factors. A minority provided analgesia, or screening for CVD risk factors, and depression. Most GPs referred to dermatology if secondary care input was required, with few referrals to specialised multidisciplinary services.

**Conclusion:**

GPs regularly diagnose and manage uncomplicated HS, but screening for important comorbidities associated with HS is not common practice.

## How this fits in

HS is associated with multiple comorbidities and high healthcare usage.^1,2^ UK guidelines for managing HS recommend first-line drugs and lifestyle interventions suitable for primary care.^2^ This study suggested GPs focus on treating skin disease and lifestyle factors. Tackling broader impacts of HS, including pain and psychological effects, could improve patients’ outcomes.

## Introduction

HS is a chronic inflammatory skin disease, with a prevalence of around 1% in Western populations.^3,4^ It often presents as recurring ‘boils’ in groins, breasts, and axillae, which fail to heal with short courses of antibiotics, but persist as painful scarring nodules and chronically discharging skin tunnels (sinuses).^5^ Many of those affected by HS have adverse lifestyle factors, particularly smoking and obesity, and it is often associated with multiple comorbidities including depression, CVD, and type two diabetes.^3,6,7^ People living with HS are typically young to middle-aged adults, and the condition can have profound and long-lasting effects on their employment prospects and quality of life.^8^ HS has historically been poorly recognised and under-researched, and delayed diagnosis and suboptimal treatment have been noted in many studies worldwide.^1,9^ However, most patients present with characteristic features, and a firm diagnosis can usually be made in the presence of a typical history and clinical findings (Box 1). Primary care practitioners are ideally placed to diagnose and treat HS in its early stages. They also have the skills to assess patients for the excess cardiovascular risk associated with HS,^7^ and to support patients in making lifestyle changes and coping with the psychosocial effects of the disease.^6^ Despite this potentially important role, there has been little research into the area of primary care practitioners’ management of HS in the UK. This study aimed to understand current practice in treating HS in primary care.

Box 1 **Diagnostic features of hidradenitis suppurativa^8^**

- Typical lesions: inflamed nodules, discharging abscesses, chronic sinus tracts, rope-like scars, and comedones.
- Typical sites: groin and axillae are commonest, but breast, neck, lower abdomen, and perineum are also recognised sites.
- Typical course: skin lesions recurring or non-resolving at the same sites, despite standard short antibiotic courses. At least two lesions in the past 6 months or a lifetime history of at least five lesions.

This is one of a series of surveys of different professional groups that aimed to inform the delivery of the Treatment of Hidradenitis Suppurativa Evaluation Study (THESEUS). THESEUS has been funded by the National Institute for Health Research to document current practice in treating HS, and assess the feasibility of future randomised controlled trials of HS treatments.^10,11^ By mapping the primary care management of HS and the pathways from primary to secondary care, the study aimed to establish which treatments patients were likely to have had before reaching secondary care, and to which specialties they were being referred.

The aim of the survey was to explore the following research questions:

- What are the current drug and non-drug management options recommended by UK GPs to people with HS?
- What are the common referral pathways of patients with HS from primary to secondary care in the UK?

## Method

### Questionnaire

A custom-built online open survey^12^ was designed using the REDCap secure web application. This was anonymised, but multiple entries by individuals were prevented by requiring a ‘return code’ for revisiting the survey. The list of questions in the survey are shown in Supplementary Table S1. Owing to the convenience nature of the sample, the initial part of the survey explored characteristics of responders that may influence their experience in treating HS. Questions then explored which interventions GPs would offer in primary care before considering secondary care referral. The interventions were based on recommendations of the British Association of Dermatologists’ guidelines for the treatment of HS, which included: extended courses of oral tetracycline-type antibiotics; 10-week courses of clindamycin and rifampicin; lifestyle advice; and surgical interventions.^2^ Questions also looked at GPs’ choice of secondary care specialty for referral of patients with HS and which factors determined their choice.

The option to add a free-text comment was offered for each question.

### Population and setting

This was a cross-sectional survey of UK GPs including those with, and without, a stated special interest in dermatology, selected using convenience sampling. The electronic link to the questionnaire was active between May and September 2018.

Three methods were used to distribute the survey:

1. The PCDS distributed an electronic link to the survey to their 4000 members by email.
2. An electronic link to the survey was distributed to all 247 GPs in NHS Forth Valley in Central Scotland via group email and GPs were encouraged to share the link with colleagues.
3. Members of the research group distributed the survey link to GPs in their area of the UK who might be interested in completing the survey.

### Data analysis

Sample size was not pre-specified owing to convenience sampling, but it was aimed to get at least 100 responses. The quantitative data were analysed with descriptive statistics.

## Results

### Descriptive statistics of GP survey responders

A total of 135 GPs responded to the invitation and 134 answered survey questions. Ninety-seven per cent (*n* = 130) of responders completed all questions, and the four partially completed surveys were included in the analysis. Responses from all three sources were analysed together. Percentages were calculated according to the number of responses for each question, not the overall sample size. Twenty-one free-text comments were submitted, which were mostly brief and insufficient for analysis.

Owing to the sampling method used, most English, Irish, and Welsh GP responders were members of the PCDS and so had a dermatology special interest, whereas the Scottish responders were largely GPs in NHS Forth Valley who reported no particular interest or experience in dermatology. The geographical distribution and special interest status of responders is shown in Table 1. Eighty responders (60%) reported a special interest in dermatology, including 53 who stated they had some form of postgraduate experience or training in dermatology, and 38 holding a formal ‘GP with special interest’ post.

**Table 1.** Proportion of GPs with dermatology special interest and location of practice

Location of GPparticipants	**No dermatology special interest, *n***	**Dermatology special** **interest, *n***	**Total, *n***
England	7	58	65
Northern Ireland	0	2	2
Scotland	46	16	62
Wales	2	4	6
**Total**	**55**	**80**	—

Survey responders were predominately experienced GPs, with 90% (*n* = 120) having worked for >5 years in general practice (see Supplementary Table S2). Most worked in medium-to-large sized practices, with 40% (*n* = 53) of the sample having a list size of 5000–10 000 patients and 36% (*n* = 48) having >10 000 patients (see Supplementary Table S3). Only one responder said they had never had a patient with HS, and 70% (*n* = 94) had seen at least one patient with HS in the month preceding the survey (Table 2).

**Table 2.** Number of patients with HS seen by GPs in month before survey

**HS patients **seen in past month, *n*****	**Total, *n***	**Total, %**
0	40	30
1	45	34
2	32	24
3–5	14	10
>5	3	2
**Grand total**	**134**	**100**

### Confidence in diagnosing and treating HS in primary care

The survey results showed that most GPs in the sample considered themselves to be confident in diagnosing HS whether or not they had a dermatology special interest. Overall 94% reported confidence in diagnosis with 53% (*n* = 71) stating they required no secondary care input to diagnose the condition, and 40% (*n* = 54) saying they were confident but might refer for confirmation on occasion (data not shown). Confidence levels were also high in the ongoing management of HS, with 76% (*n* = 42) of GPs with no special dermatology interest prepared to manage uncomplicated HS in primary care, either independently or following specialist diagnosis, and 94% (*n* = 75) of special interest GPs. GPs working in practices with a list size >10 000 (*n* = 48) were most likely to report confidence in managing uncomplicated HS wholly within primary care (*n* = 30, 63%). Confidence levels declined with smaller practice size, with 42% (*n* = 22) of the 53 GPs in medium-sized practices (5000–10 000) and 30% (*n* = 6) of the 20 GPs in small practices (<5000) reporting this level of confidence. Of responders who had been qualified as GPs <5 years, 38% (*n* = 5) reported confidence in managing uncomplicated HS independently, compared with 47% (*n* = 8) of those qualified 5–10 years, 56% (*n* = 20) of those 10–20 years, and 47% (*n* = 32) of those >20 years post-qualification (see Supplementary Tables S2 and S3).

### Current practice in treating hidradenitis suppurativa

**Figure 1.**
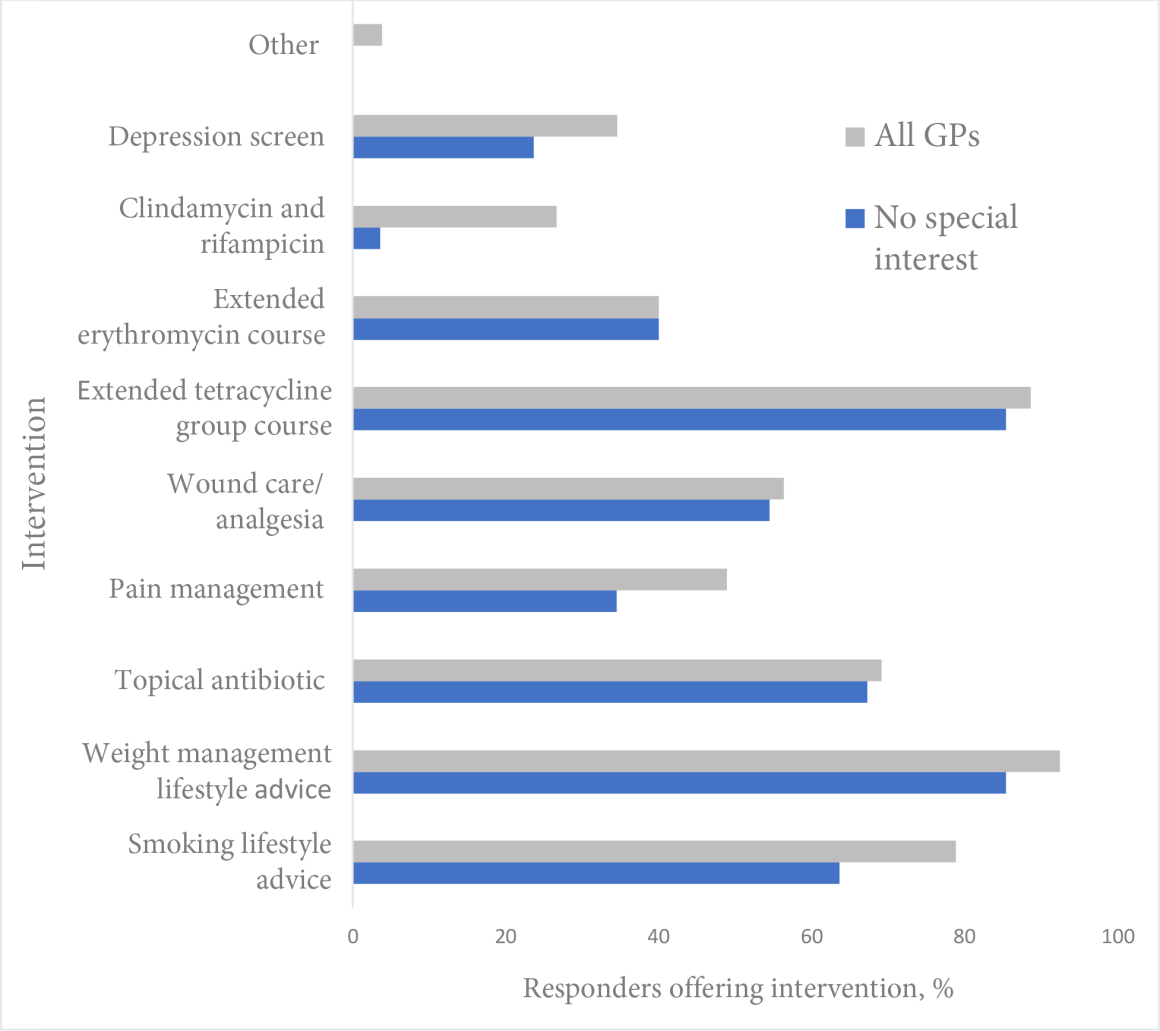
Medical interventions for hidradenitis suppurativa. GPs choice of options for what they would try before referral, comparing overall GP sample with subgroup of GPs with no dermatology special interest. Other = free-text replies: metformin, ‘botox’, isotretinoin, comorbidity screening.

The majority of GPs reported that they would offer advice about smoking (*n* = 105, 78%) and weight management (*n* = 124, 93%) before considering specialist referral. Other aspects of holistic care were less commonly offered, including analgesia (*n* = 65, 49%), wound care (*n* = 75, 56%), cardiovascular risk assessment (*n* = 62, 46%, data not shown) and depression screening (*n* = 46, 34%) (Figure 1).

Eighty-eight per cent (*n* = 118) of GP responders reported treating HS patients with a 3-month course of tetracycline-type oral antibiotics before specialist referral, including 84% (*n* = 46) of GPs without a dermatology special interest (Table 3). Guidelines recommend a course of combined clindamycin and rifampicin following failure of oral tetracycline therapy.^2^ Only 26% (*n* = 35) of the GP sample would consider prescribing this, and only 4% (*n* = 2) of non-specialist GPs would do so (Figure 1).

**Table 3.** Key interventions offered to patients with HS by GPs before specialist referral

Intervention offered	GP dermatology special interest, *n* (%)
Special interest	No special interest
Weight management lifestyle advice	77 (96)	47 (85)
Tetracycline antibiotic, 3 months	72 (90)	47 (85)
Smoking lifestyle advice	70 (88)	35 (64)
Topical antibiotic	55 (69)	37 (67)
Wound care	46 (57)	30 (55)
Pain management	46 (57)	19 (35)
Depression screen	33 (41)	13 (24)

A minority of GPs offered minor surgical interventions for HS in primary care: 31% (*n* = 41) said they might incise and drain an acutely inflamed lesion, and 7% (*n* = 10) reported that they would excise a chronic non-resolving inflammatory nodule.

### Referral to secondary care

**Figure 2.**
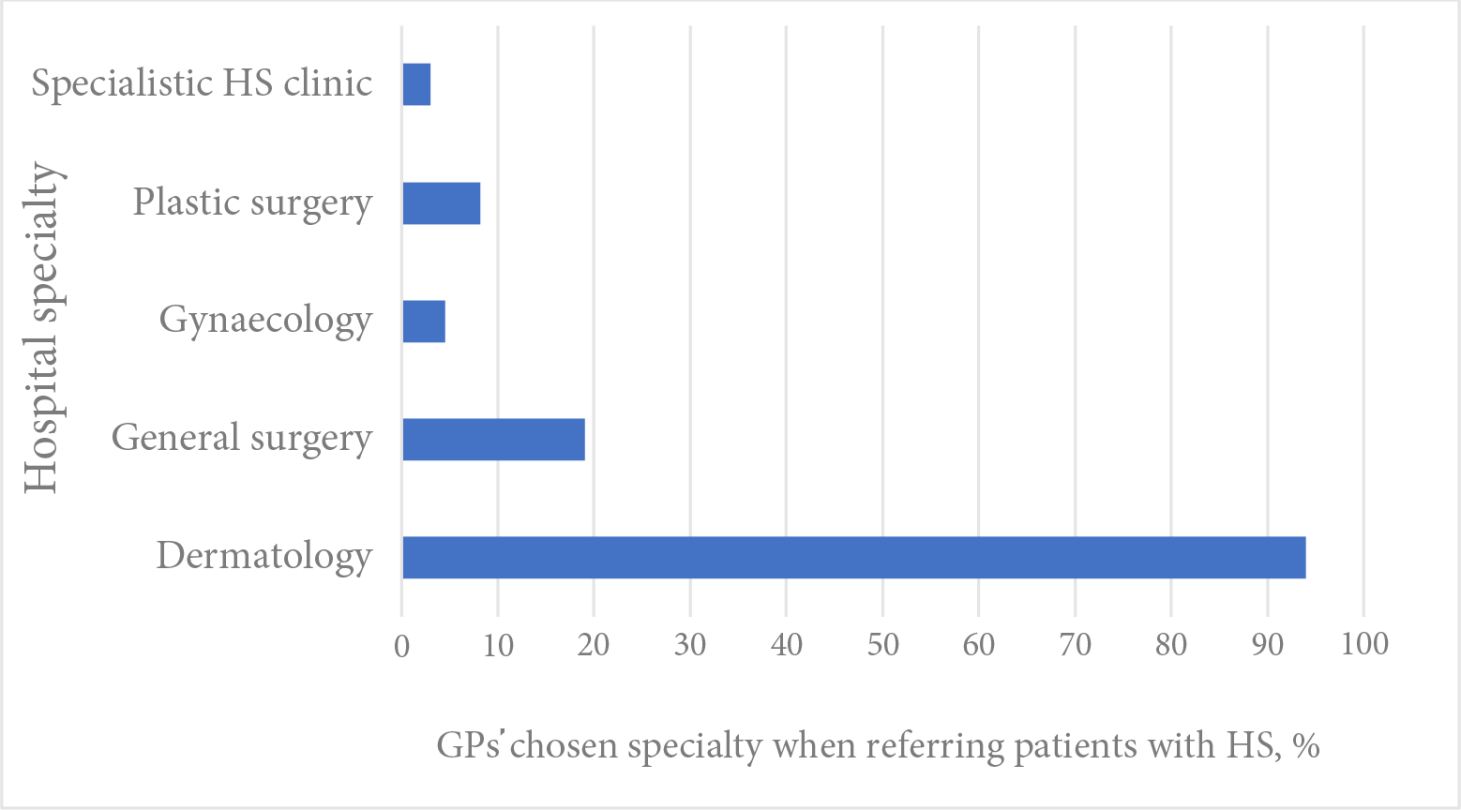
GPs’ choice of specialty when referring patients with hidradenitis suppurativa (HS) to secondary care

Over 90% (*n* = 126) of the GP sample reported referral of patients with HS to dermatology; however, 19% (*n* = 25) would still choose to refer some patients to general surgery (Figure 2). A number of factors had a strong influence on choice of referral specialty: 92% (*n* = 123) said that disease severity was ‘very’ important or ‘somewhat’ important in determining choice, with one GP commenting that they would usually refer a patient with a ‘particularly large abscess’ to surgery. Eighty per cent (*n* = 107) felt the patient’s previous care by a specialty would influence re-referral to that specialty. HS affecting a particular body site would be ‘very’ or ‘somewhat’ important in choice of referral specialty for 80% of GPs (*n* = 107); for example, gynaecology for vulval HS. The patient’s preference for a particular specialty was considered an important determinant of the referral pathway by 66% of GPs (*n* = 89), the existence of an agreed local patient pathway for patients with HS by 77% (*n* = 103); and the presence of a local clinician with a special interest in HS by 78% (*n* = 104). Only 3% (*n* = 4) of GPs reported referring patients to a specialised multidisciplinary service for HS.

## Discussion

### Summary

To the authors’ knowledge, this study is the first to look at UK GPs' self-reported practice in treating HS. Most GPs in the sample reported high levels of confidence in diagnosis and initial management of HS treatments, perhaps reflecting the high proportion of responders with a special interest in dermatology.

Initial medical management of the skin manifestations of HS was largely compliant with UK guidance,^2^ but suboptimal management of pain, psychosocial aspects, and comorbidity screening suggests an educational need among UK GPs, particularly those in smaller practices or in the early years of their career. This was particularly striking given the high levels of reported confidence in managing the condition.

### Strengths and limitations

This study breaks new ground in asking UK GPs directly about their management of HS. It is not possible to ascertain from this data how much this reflects GPs' actual practice, but it does show their awareness of recommended HS management.

The preponderance among responders of experienced GPs (90% in practice for at least 5 years) and GPs working in larger practices of at least 5000 patients (75%), may have made it more likely that they would have experience of seeing patients with HS.

Owing to the convenience sampling method, sampling bias is possible and responders may not be representative of the population of UK GPs. Over half of the sample had a special interest in dermatology so, where relevant, differences in responses between those with and without a special interest are highlighted (Figure 1, Table 3). However, practitioners who had no knowledge of HS may have been less likely to respond to the invitation to take the survey.

The sample included GPs throughout the UK but geographical differences in results are confounded by the predominance of non-specialist GPs in the Scottish responders.

### Comparison with existing literature

This study looked at self-reported rather than objectively measured practice. Previous research into HS management in primary care has included a 2016 UK study, drawing on a large database of anonymised GP patient records, which demonstrated that patients with HS are frequent attenders at primary care and often experience significant delays in receiving a diagnosis.^1^ A 2015 study that assessed Danish and Belgian GPs’ knowledge of HS found important deficiencies in their knowledge of the condition.^13^ In the present study, most GPs felt they were able to diagnose HS confidently, suggesting either the sample was atypical, or GPs may be unaware of delayed or missed diagnosis in a proportion of patients.

Comorbidities add significantly to the disease burden of HS; for example, several studies have documented high levels of CVD^14^ and sudden cardiac death associated with HS**^7^**. Anxiety, depression, and suicidality are also more common in people with HS than in the general population.^15^ Pain may be experienced acutely owing to inflamed lesions, and also have a chronic aspect, and has been found to be one of the most debilitating aspects of HS,^16^ with the HS Priority Setting Partnership process concluding that effective analgesia was one of the top 10 priorities.^17^

### Implications for research and practice

Recent advances in the treatment of HS have focused on interventions for people with more severe forms of HS, and biologic drugs have been life-changing for some patients.^18^ However, many patients with HS have milder forms of the condition that never progress to this severity. People with milder forms of HS can often be managed effectively and holistically in primary care, with appropriate support where required from specialists in secondary care.^2^ Prompt and effective treatment and lifestyle advice early in the course of the condition may prevent some patients progressing to more severe disease requiring second-line treatments, and extensive surgery for disfiguring scars and skin tunnels.^2^ Management of pain, anxiety, and depression will improve quality of life and patients’ ability to manage their condition.

UK guidelines recommend that surgical intervention, particularly localised excisions, should be undertaken as part of an overall management plan for patients with HS rather than as an isolated intervention, as the disease is very likely to recur.^2^ The present study's data suggest that some patients are still being referred for localised surgical excisions directly from general practice, and it is unclear whether their disease is being controlled systemically before the referral, as is recommended. Local referral pathways for patients with HS might be helpful in ensuring patients have a dermatological assessment of the overall disease burden before surgical intervention. Very few GPs in the sample currently refer to a multidisciplinary HS clinic as recommended by the British Association of Dermatologists guidelines,^2^ possibly reflecting the fact that few hospitals offer this service in the UK.

In conclusion, dermatology services are not configured to offer support for lifestyle changes, or manage comorbidities common in HS, such as CVD, diabetes, and depression, which are areas where primary care has expertise. Primary care itself is at a crisis point in managing workload; however, people with HS are already being seen frequently in primary care; for example, on average 8.9 times per patient annually.^1^ The interventions required at early stages of the disease, such as oral tetracycline-type drugs, support for lifestyle change, pain and wound management, and screening for depression and cardiovascular risk, are ones GPs are experienced in using. GPs in this study, with and without a special interest in dermatology, were confident in initiating first-line management of HS in primary care and offering lifestyle advice. Targeted education for primary care might be useful in also raising awareness of common comorbidities and complications of HS, and the importance of managing these to improve quality of life.

Future research using primary care electronic prescribing data on drug therapies prescribed for HS, and surveys of regional referral pathways, would help to describe UK primary care management of HS more fully.

## References

[1] Bewley A, Malcolm B, Shah P (2016). Main plenary sessions. The primary care management of hidradenitis suppurativa in the UK: an evaluation of patient pathways and healthcare resource use using the Health Improvement Network database. Br J Dermatol.

[2] Ingram JR, Collier F, Brown D (2019). British Association of Dermatologists guidelines for the management of hidradenitis suppurativa (acne inversa) 2018. Br J Dermatol.

[3] Revuz JE, Canoui-Poitrine F, Wolkenstein P (2008). Prevalence and factors associated with hidradenitis suppurativa: results from two case-control studies. J Am Acad Dermatol.

[4] Ingram JR, Collins H, Atkinson MD, Brooks CJ (2020). The prevalence of hidradenitis suppurativa is shown by the Secure Anonymised Information Linkage (SAIL) Databank to be one per cent of the population of Wales. Br J Dermatol.

[5] Revuz JE, Jemec GBE (2016). Diagnosing hidradenitis suppurativa. Dermatol Clin.

[6] Kouris A, Platsidaki E, Christodoulou C (2016). Quality of life and psychosocial implications in patients with hidradenitis suppurativa. Dermatology.

[7] Egeberg A, Gislason GH, Hansen PR (2016). Risk of major adverse cardiovascular events and all-cause mortality in patients with hidradenitis suppurativa. JAMA Dermatol.

[8] Matusiak Łukasz, Bieniek A, Szepietowski JC (2010). Hidradenitis suppurativa markedly decreases quality of life and professional activity. J Am Acad Dermatol.

[9] Saunte DM, Boer J, Stratigos A (2015). Diagnostic delay in hidradenitis suppurativa is a global problem. Br J Dermatol.

[10] Cardiff University THESEUS. https://www.cardiff.ac.uk/centre-for-trials-research/research/studies-and-trials/view/theseus.

[11] National Institute for Health Research (2020). Treatment of Hidradenitis Suppurativa Evaluation Study (THESEUS). https://fundingawards.nihr.ac.uk/award/17/98/01.

[12] Eysenbach G (2004). Improving the quality of web surveys: the Checklist for Reporting Results of Internet E-Surveys (CHERRIES). J Med Internet Res.

[13] Benhadou F, Theut Riis P, Njimi HH (2015). Hidradenitis suppurativa in general practice: a pilot study. J Gen Pract.

[14] Tzellos T, Zouboulis CC, Gulliver W (2015). Cardiovascular disease risk factors in patients with hidradenitis suppurativa: a systematic review and meta-analysis of observational studies. Br J Dermatol.

[15] Patel KR, Lee HH, Rastogi S (2020). Association between hidradenitis suppurativa, depression, anxiety, and suicidality: a systematic review and meta-analysis. J Am Acad Dermatol.

[16] Patel ZS, Hoffman LK, Buse DC (2017). Pain, psychological comorbidities, disability, and impaired qualify of life in hidradenitis suppurativa. Curr Pain Headache Rep.

[17] Ingram JR, Abbott R, Ghazavi M (2014). The hidradenitis suppurativa priority setting partnership. Br J Dermatol.

[18] Kimball AB, Kerdel F, Adams D (2012). Adalimumab for the treatment of moderate to severe hidradenitis suppurativa: a parallel randomized trial. Ann Intern Med.

